# Activity-Guided Isolation of Antioxidant Compounds from *Rhizophora apiculata*

**DOI:** 10.3390/molecules170910675

**Published:** 2012-09-06

**Authors:** Mingzhe Gao, Hongbin Xiao

**Affiliations:** Dalian Institute of Chemical Physics, Chinese Academy of Sciences, Dalian 116023, China

**Keywords:** *Rhizophora apiculata*, antioxidant activity, total phenolic content, lignan, flavonol

## Abstract

*Rhizophora apiculata* (*R. apiculata*) contains an abundance of biologically active compounds due its special salt-tolerant living surroundings. In this study, the total phenolic content and antioxidant activities of various extract and fractions of stem of *R. apiculata* were investigated. Results indicated that butanol fraction possesses the highest total phenolic content (181.84 mg/g GAE/g dry extract) with strongest antioxidant abilities. Following *in vitro* antioxidant activity-guided phytochemical separation procedures, lyoniresinol-3α-*O*-β-arabinopyranoside (**1**), lyoniresinol-3α-*O*-β-rhamnoside (**2**), and afzelechin-3-*O*-L-rhamno-pyranoside (**3**) were separated from the butanol fraction. These compounds showed more noticeable antioxidant activity than a BHT standard in the DPPH, ABTS and hydroxyl radical scavenging assays. HPLC analysis results showed that among different plant parts, the highest content of **1**–**3** was located in the bark (0.068%, 0.066% and 0.011%, respectively). The results imply that the *R. apiculata* might be a potential source of natural antioxidants and **1**–**3** are antioxidant ingredients in *R. apiculata*.

## 1. Introduction

Oxygen-centered free radicals and other reactive oxygen species (ROS) can be generated as byproducts during oxidative progresses of living organisms [1]. Many human diseases, including accelerated aging, cancer, inflammation, cardiovascular and neurodegenerative disease and, are linked to excessive amounts of free radicals [2]. Antioxidants are necessary to supplement the natural antioxidant defenses of the body to cure these diseases. However, the synthetic antioxidants might be unsafe, therefore, more attention is being paid to searching for natural antioxidants from plants to prevent oxidative damage [3].

Mangrove plants are composed of a large group of different salt-tolerant plants growing in tropical and subtropical intertidal estuarine areas. These plants, constantly subjected to tidal flushing with the ability to live in salt water, have specially adapted their own morphological structures and physiological mechanisms to their harsh natural surroundings [4]. *Rhizophora apiculata* (*R. apiculata*), a member of the mangrove plants, has been used by the local people for ages as a sterilizing agent, deodorizer and growth promoting agent [5]. Triterpenes and diterpenoids were isolated from this plant and phenolic compounds were found in the pyroligneous acid of *R. apiculata*[6,7,8]. It was reported that the bark extract of *R. apiculata* showed antioxidant activities [9,10,11]. However, the compounds responsible for the antioxidant ability in crude plant have not previously investigated. In this study, an activity-guided phytochemical isolation method was proposed to prepare the antioxidant compounds from extracts of *R. apiculata* under direction of *in vitro* antioxidant tests.

## 2. Results and Discussion

### 2.1. Antioxidant Activities of Crude Extract and Fractions

**Figure 1:**
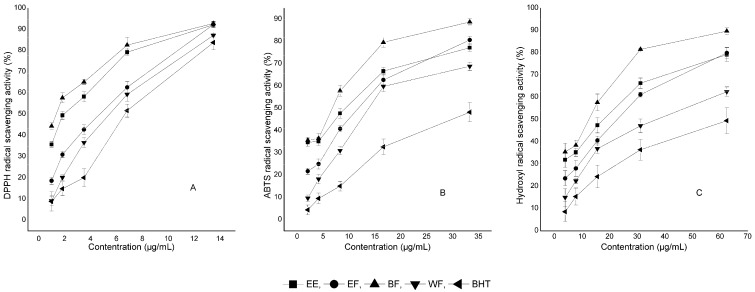
Antioxidant activities of extract and fractions of *R. apiculata.*(**A**) DPPH; (**B**) ABTS^•+^; (**C**) Hydroxyl radicals.

The DPPH radical assay is a suitable model for estimating total antioxidant potential of antioxidants [12]. Figure 1A shows the dose-response curves of the DPPH radical scavenging activities of crude extract and fractions from *R. apiculata*. All samples have antioxidant activity against DPPH and the reducing power increased as the sample concentration increased from 2.09 to 33.34 µg/mL. The butanol fraction (BF) exhibits the highest scavenging activity of 89.3% at the concentration of 33.34 µg/mL, whereas the ethanol extract (EE), ethyl ester fraction (EF) and water fraction (WF) show 77.9%, 79.9% and 67.21% at the same concentration, respectively. IC_50_ values, defined as the concentration with 50% activity, were calculated for comparison. The IC_50_ values of scavenging activities for EE, EF, BF, WF and positive control BHT were 13.56 ± 1.79, 19.31 ± 1.56, 9.68 ± 1.86, 23.72 ± 1.94 and 52.20 ± 1.57 μg/mL, respectively. According to these IC_50_ values, the DPPH radical scavenging ability was found in the order of BF > EF > EE > WF > BHT (*P* < 0.05).

ABTS is another widely used synthetic radical for both the polar and non-polar samples [13]. The ABTS^•+^ scavenging abilities of the crude extract and fractions of *R. apiculata* were plotted in Figure 1B. EE, EF and BF exhibit a maximum scavenging activity of above 90% at the concentration of 13.33 μg/mL while WF shows 87.22% inhibition ability at the same concentration. The IC_50_ values of the scavenging activities of EE, EF, BF, WF and BHT were 1.71 ± 0.39, 3.01 ± 0.75, 1.26 ± 0.05, 4.32 ± 0.96 and 9.63 ± 0.15 μg/mL, respectively. The order of ABTS radical scavenging ability was BF > EF > EE > WF> BHT (*P* < 0.05).

Hydroxyl radicals are highly reactive and short-lived species causing damage to virtually all adjacent biomolecules [14]. Its radical scavenging abilities of extract/fractions of *R. apiculata* were investigated and are shown in Figure 1C. Like in the DPPH and ABTS assays, the hydroxyl radical scavenging abilities increase with the increased concentration of the test samples. The IC_50_ values of EE, EF, BF, WF and BHT were 13.57 ± 1.59, 17.93 ± 1.51, 9.07 ± 0.99, 33.59 ± 1.66 and 45.58 ± 2.14 µg/mL, respectively. According to these IC_50_ values, the order of hydroxyl radical scavenging abilities is BF > EE >EF > WF > BHT (*P* < 0.05).

### 2.2. Total Phenolic Content

Phenolic compounds are a large group of phytochemical components widespread in the plant kingdom and characterized by having at least one aromatic ring with one or more hydroxyl groups attached which directly contribute to the antioxidant properties [15]. Therefore, it is important to evaluate the total phenolic in the crude extract and fractions of the *R apiculata*. The total phenolic content was expressed in milligrams equivalents of gallic acid per gram of each fraction. The amount of phenolic compounds among different fractions were in the order of BF (181.84 ± 2.56 mg/g) > EE (127.81 ± 4.5 mg/g) > EF (121.16 ± 2.57 mg/g) > WF (106.88 ± 9.87 mg/g), results in our study show that the extent of antioxidant activity of fraction is in accordance with the total phenolic content.

### 2.3. Separation of Compounds

According to the above results, the BF contains the largest amount of phenolic compounds and exhibits the strongest radical scavenging activities. Therefore the BF was further fractionated to identify the compounds responsible for the antioxidant activity. Compounds **1**–**3** were obtained from BF and identified as lyoniresinol-3α-*O*-β-arabinopyranoside, lyoniresinol-3α-*O*-β-rhamnoside and afzelechin-3-*O*-L-rhamnopyranoside by comparing their NMR data with literature data [16,17,18]. To the best of our knowledge, it is first report of the separation of lignan and flavonol compounds from *R. apiculata*.

### 2.4. Activity and Quantity Analysis of Isolated Compounds

Lignans and flavonols are a group of compounds which show several biological activities. In our experiment, all separated compounds showed remarkable antioxidant activity and the IC_50_ values were listed in Table 1. The results indicated that compounds **1**–**3** were active ingredients in *R. apiculata*.

**Table 1:** Antioxidant activity of compounds isolated from *R. apiculata.*

Compound	IC_50_ (μg/mL)		
	DPPH	ABTS^•+^	OH
Lyoniresinol-3α-*O*-β-arabinopyranoside	2.06	1.64	5.83
Lyoniresinol-3α-*O*-β-rhamnoside	2.64	2.09	9.07
Afzelechin-3-rahmnoside	2.26	1.96	7.05
BHT	55.20	9.63	45.58

**Figure 2:**
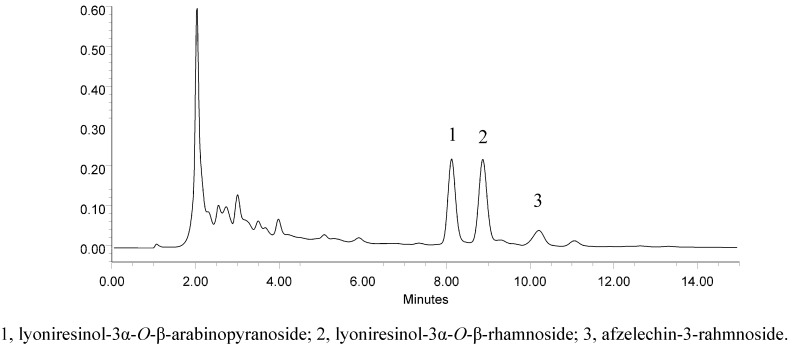
Chromatogram of crude extract of *R. apiculata*.

*R. apiculata* is a protected marine plant in China. To reasonably utilize the mangrove plant resource, a HPLC method was developed to study the distribution of the three compounds in different parts of *R. apiculata*. Excellent separation was achieved under the optimized chromatographic conditions and the HPLC chromatogram of the crude extract is shown in Figure 2. Results (Table 2) showed the highest content was located in the bark and lowest content was in leaves, suggesting that bark of *R. apiculata* was a better resource of **1**–**3** than the other two parts of the plant.

**Table 2:** Content of compounds in different part of *R. apiculata*.

Compound	Content (%)		
	twig	leaf	bark
Lyoniresinol-3α-*O*-β-arabinopyranoside	0.047	0.012	0.068
Lyoniresinol-3α-*O*-β-rhamnoside	0.059	0.016	0.066
Afzelechin-3-rahmnoside	0.010	0.006	0.011

## 3. Experimental

### 3.1. Plant Material and Chemicals

*R. apiculata* was collected from Hainan province of China in June 2006 and identified by Prof. Shixiang Bao. A voucher specimen was deposited at Dalian Institute of Chemical Physics, Chinese Academy of Sciences. 1,1′-Diphenyl-2-picrylhydrazyl (DPPH), 2,2′-azinobis-(3-ethylbenzo-thiazoline-6-sulphonic acid) diammonium salt (ABTS), and butylated hydroxytoluene (BHT) were obtained from Sigma Chemical Co. (St. Louis, MO, USA). Gallic acid was obtained from National Institute for the Control of Pharmaceutical and Biological Products (Beijing, China). All other analytical grade chemicals were obtained from Kemio Chemical Co. (Tianjin, China).

### 3.2. Extraction and Chemical Isolation

Dried stems of *R. apiculata* (4.5 kg) were refluxed twice with 15 L ethanol for 3 h. The collected extract solution was concentrated and the final yield of ethanol extract was 281 g. A portion of this EE (270 g) was fractionated successively with ethyl acetate and butanol. After removing the solvent, three fractions were obtained. The yields of EF, BF and WF were 48.0, 71.8 and 110 g, respectively. BF was subjected to macrospore resin column chromatography and further separated with preparative HPLC. Compounds **1** (100 mg), **2** (95 mg) and **3** (9.7 mg) were obtained after evaporating the solvent.

### 3.3. Antioxidant Activity

#### 3.3.1. DPPH free Radical Scavenging Assay Activity

The DPPH free radical scavenging capability was determined with the method described previously by Kaur [19]. Samples (50 µL) at various concentrations was mixed with 0.1 nM DPPH solution (2,950 µL) and the absorbance measured at 517 nm after 30 min. The capability to scavenging the DPPH radical was calculated using the following equation: 


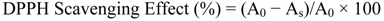


where A_0_ is the absorbance of the blank reaction and A_s_ is the absorbance of the sample.

#### 3.3.2. ABTS Scavenging Activity

ABTS scavenging activity of the sample was investigated by the method of Re [13] with some modifications. The ABTS solutuion was prepared by mixing 7.4 mM ABTS diammonium salt solution and 2.6 mM potassium persulfate solution in equal volume. The mixture was reacted for 12 h in the dark and then diluted to obtain an absorbenece of 0.7 at 734 nm. Sample (20 µL) was mixed with ABTS solution (2,980 µL) and the absorbance of the mixture at 734 nm was monitored after 5 min. The scavenging ability of antioxidants was calculated according to the same equation as that in the DPPH assay.

#### 3.3.3. Hydroxyl Radical Scavenging Ability Assay

The hydroxyl radical scavenging effect was evaluated based on the Fenton reaction described by Yu [20]. Sample (0.5 mL) was mixed with ferrous chloride (0.5 mL, 3 mM), 1,10-phenanthroline (0.5 mL, 3 mM), phosphate buffer (2 mL, 2.5 mM, pH = 7.4) to prepare the mixture. 0.1% Hydrogen peroxide (0.5 mL) was added to the mixture to initiate the reaction. After 30 min incubation at 37 °C, the absorbance of the mixture was measured at 560 nm. Hydroxyl radical scavenging ability was expressed by the following equation:


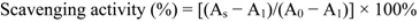


where A_0_ is the absorbance of control without test sample and H_2_O_2_, A_1_ is the absorbance of control without test sample and A_s_ is the absorbance of the test sample.

### 3.4. Total Phenolic Content

The amount of total phenolics was measured by the Folin-Cioaclteu method [21]. Folin-Ciocalteu reagent (0.5 mL) was added to a 1 mL sample and the mixture was kept for 5 min before the addition of 20% Na_2_CO_3_ (2 mL). The solution was allowed to stand for 10 min and then measured at 730 nm.

### 3.5. HPLC Analysis of Lignans

Bark, leaf and twig of *R. apiculata* were analyzed to study the distribution of the compounds in plant. Samples (1 g) were ultrasonically extracted with methanol (10 mL) and the extract solvent was used as sample. The separation was performed on an Inertsil ODS2 column (250 × 4.6 mm, 5 µm). The mobile phases consisted of acetonitrile (A) and water. Gradient elution was started with 15% A and ascended to 20% A in 20 min at a flow rate of 1.0 mL/min. The injected volume was 10 µL and absorbance value was measured at 207 nm.

### 3.6. Statistical Analysis

Datas were reported as mean ± SD from triplicate determinations. Statistical analysis was performed with Student’s t-test. A difference was considered statistically significant, when *P* < 0.05.

## 4. Conclusions

An activity-guided phytochemical isolation method was used to study the active compounds in *R. apiculata*. The results indicated that *R. apiculata* stem extract/fractions exhibit excellent radical scavenging ability in all assays employed and BF was the most active fraction among them. Phytochemical investigation of the BF led to the separation of compounds **1**–**3** which were separated from *R. apiculata* for the first time. Radical scavenging assays indicated all compounds had stronger antioxidant capacity than the positive control BHT. HPLC analysis results showed that among different plant parts, the highest content of **1**–**3** was located in the bark. Overall, *R. apiculata* is a promising source of natural antioxidants and **1**–**3** are antioxidant ingredients in *R. apiculata*.
